# Hit discovery of potential CDK8 inhibitors and analysis of amino acid mutations for cancer therapy through computer-aided drug discovery

**DOI:** 10.1186/s13065-024-01175-6

**Published:** 2024-04-13

**Authors:** Raziye Aghahasani, Fereshteh Shiri, Hossein Kamaladiny, Fatemeh Haddadi, Somayeh Pirhadi

**Affiliations:** 1 Department of Biology, University of Zabol, Zabol, Iran; 2 Department of Chemistry, University of Zabol, Zabol, Iran; 3 Medicinal and Natural Products Chemistry Research Center, Shiraz University of Medical Sciences, Shiraz, Iran

**Keywords:** Cyclin-dependent kinase, Cancer, Mutation, Molecular dynamic, Virtual screening

## Abstract

Cyclin-dependent kinase 8 (CDK8) has emerged as a promising target for inhibiting cancer cell function, intensifying efforts towards the development of CDK8 inhibitors as potential cancer therapeutics. Mutations in CDK8, a protein kinase, are also implicated as a primary factor associated with tumor formation. In this study, we identified potential inhibitors through virtual screening for CDK8 and single amino acid mutations in CDK8, namely D173A (Aspartate 173 mutate to Alanine), D189N (Aspartate 189 mutate to Asparagine), T196A (Threonine 196 mutate to Alanine) and T196D (Threonine 196 mutate to Aspartate). Four databases (CHEMBEL, ZINC, MCULE, and MolPort) containing 65,209,131 molecules have been searched to identify new inhibitors for CDK8 and its single mutations. In the first step, structure-based pharmacophore modeling in the Pharmit server was used to select the compounds to know the inhibitors. Then molecules with better predicted drug-like molecule properties were selected. The final filter used to select more effective inhibitors among the previously selected molecules was molecular docking. Finally, 13 hits for CDK8, 11 hits for D173A, 11 hits for D189N, 15 hits for T196A, and 12 hits for T196D were considered potential inhibitors. A majority of the virtual screening hits exhibited satisfactorily predict pharmacokinetic characteristics and toxicity properties.

**Supplementary Information:**

The online version contains supplementary material available at 10.1186/s13065-024-01175-6.

## Introduction

In 2020, cancer was identified as the primary or secondary cause of death in 112 nations, claiming the lives of ten million individuals before they reached the age of 70 [1, 2]. Several investigations have explored methods of treating cancer that focus on the signaling pathways within cancer cells, particularly those related to protein kinases [3, 4]. Among these kinases, cyclin-dependent kinases (CDKs) belonging to the serine/threonine kinase family play a crucial role in governing various cellular processes such as transcription, apoptosis, differentiation, nerve growth, and cell cycle regulation [5]. In terms of structure, CDK8 displays the characteristic bilobal kinase fold [6]. The N-terminal domain predominantly consists of β-sheets accompanied by two α-helices, whereas the C-terminal domain is primarily α-helical. Linking these two domains is a section referred to as the hinge. The catalytic cleft, situated between the two domains, is defined by the hinge region, housing active site residues contributed by both domains [7]. Protein kinase mutations have been identified as a leading cause of a range of human diseases, particularly those that lead to the formation of tumors [8, 9]. In the case of CDK8, a member of the CDK family, its role in regulating the cell cycle and transcription activity has garnered significant research attention in recent years [10, 11]. CDK8 has emerged as a significant target for drug therapy in various cancers, including melanoma, breast, prostate, and pancreatic cancers [12–14]. It is noteworthy that the alteration of two Aspartic acid (Asp) residues, namely D173 and D189, results in the modification of the CDK8 function. The substitution of D173 with Alanine (D173A) disables CDK8’s kinase activity, rendering it inactive. Moreover, D189N mutation has been detected in various human cancers [15–17]. On the other hand, mutations T196A and T196D have been demonstrated to enhance CDK8 activity, leading to anomalous cell proliferation and division, which could potentially facilitate the advancement and exacerbation of cancer [18]. Discovering molecules that bind to therapeutic targets of interest is an ongoing endeavor in drug development. The identification and development of Type 1 CDK8 inhibitors as potential therapeutics for cancer have been an active area of research for several years. The use of molecular modeling techniques, such as virtual screening, has been a key strategy in identifying and optimizing these inhibitors. Philip et. al. in the paper “Cyclin-Dependent Kinase 8: A New Hope in Targeted Cancer Therapy?” focus on cyclin-dependent kinase 8 (CDK8), which is a protein that plays a role in regulating gene expression and has been implicated in various cancers [20]. CDK8 is abundantly present in super-enhancer (SE) regions of tumors, where it operates as a component of the mediator complex, which is generally known to act as a transcriptional coactivator. However, inhibiting CDK8 can either boost or lower SE-linked gene transcription, depending on the circumstances. For example, Cortistatin A, a natural compound that specifically inhibits CDK8, can promote the expression of SE-related genes and trigger cell death in acute myeloid leukemia [30]. Sorafenib is an FDA-approved drug available for CDK8 [19]. Additionally, CDK8 shows promise as a target for augmenting natural killer cell-mediated tumor control [31]. Designing new inhibitors for targets with amino acid mutations is crucial for several reasons. Firstly, it can help overcome drug resistance caused by changes in the shape or function of the protein. By developing inhibitors that can effectively bind to the mutated protein, treatment options can be improved. Secondly, designing new inhibitors can enhance selectivity by specifically targeting the mutated protein, reducing off-target effects. Additionally, amino acid mutations can create new binding sites or alter protein function, offering opportunities for targeted therapeutic interventions. By exploiting these changes, new therapeutic possibilities can be explored. Overall, designing inhibitors for targets with amino acid mutations addresses drug resistance, improves selectivity, and uncovers new therapeutic opportunities, leading to more effective and targeted treatments for various diseases [20, 21]. The utilization of computer-aided drug design (CADD) methods offers several advantages in the drug development process, including the reduction of time and costs involved and an enhanced likelihood of achieving successful outcomes [22, 23]. Virtual screening (VS) has shown significant success rates in computationally screening libraries of molecules to discover hits. There are two types of computational methods employed in VS: “ligand-assisted” and “structure-based”. Ligand-assisted methods use chemical fingerprints, shape, and pharmacophore features to represent known binders while docking methods dominate the structure-based approach [24–26]. Molecular dynamics simulations that take protein flexibility into account can predict protein–ligand poses that may be overlooked in molecular docking [27]. In this study, a structurally diverse set of potent inhibitors of CDK8 were used to suggest potential hits for the design and synthesis of potent CDK8 inhibitors. This was achieved through the implementation of pharmacophore modeling, drug-like molecule analysis, and molecular docking filters for virtual screening. The structure and binding information of these inhibitors proved useful in this effort.

## Materials and methods

### Data collection

Twelve compounds that were initially created as tyrosine kinase inhibitors and had kinase activity assay values of less than one micromolar (IC_50_) were chosen as type I CDK8 inhibitors [28]. Table 1 provides information regarding the structural specifics and overall arrangement of the 12 compounds analyzed. Compound 11, which exhibits the highest level of potency, displays an IC_50_ value of 1.5 nM.

**Table 1:** Structure and IC_50_ values of type I CDK8 inhibitors

No	Name	Structure	IC_50_(nM)
1	Cortistatin A		17
2	SEL120-34A		4
3	SenexinA		280
4	SenexinB		24–50
5	CCT251545		5
6	CCT251921		2.3
7	3-Methyl-1*H*-pyrazolo(3,4-b)pyridine derivative		4.4
8	1,6-Naphthyridine derivative		5.1
9	Isoquinoline derivative		32.7
10	3-Benzylindazole derivative		53
11	6-Azabenzothiophene derivative		1.5
12	BRD6989		$\approx 500$

### Homology modeling and mutation in CDK8

**Fig. 1:**
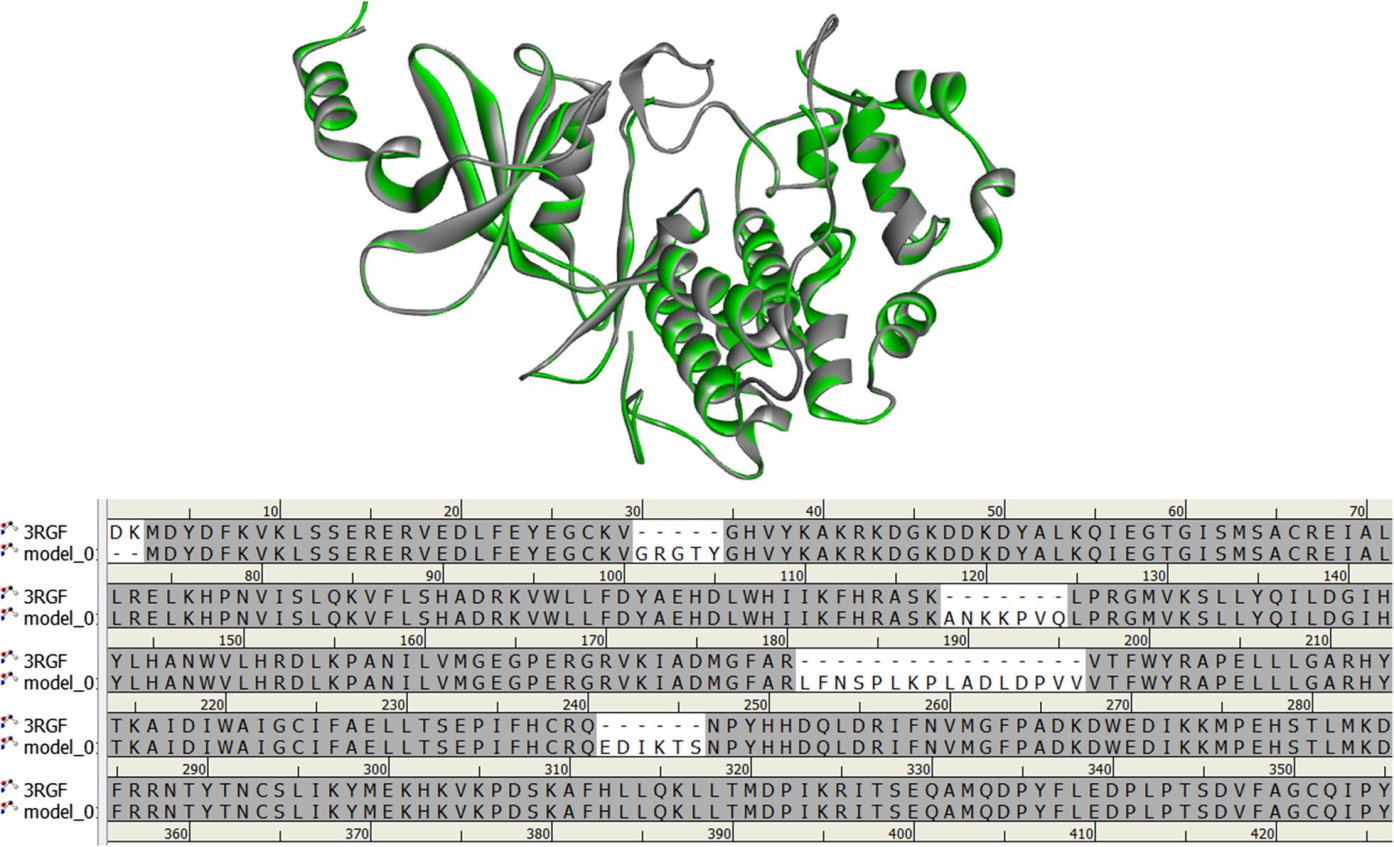
The superimposition structures of the 3RGF protein (shown in green) and the repairing Model-01 protein (shown in gray) obtained from homology modeling are displayed on the top, while the sequence alignment is shown on the bottom

Homology modeling is a valuable tool used to predict the structure of proteins in cases where experimental structures are unavailable, as well as to predict missing residues [29]. To address the missing residues in 3RGF, we employed homology modeling to reconstruct the protein structure. In order to carry out homology modeling, the mutated CDK8 protein sequence was obtained in Fasta format from the Uniport website. The Swiss model web server was utilized to import the crystal structure of CDK8 (PDB code: 3RGF) as the template. A new homology model was then created and saved in pdb format as model-01. Figure 1 displays the use of the 3RGF protein for homology modeling, with 3RGF depicted in green, Model-01 in gray, and white areas indicating the amino acids added to the model structure. We used PyMol [30] software to generate four protein structure models with single mutations of D189N, D173A, T196A, and T196D from the 3RGF structure. Then, we performed molecular dynamics simulations to determine the most stable configuration of the mutant proteins.

### Molecular docking

Molecular docking is a computational technique used to predict the binding mode of a small molecule ligand to a receptor macromolecule [31]. It involves the use of algorithms to simulate the interaction between a ligand molecule and a receptor molecule, to predict their binding affinity and to identify the most energetically favorable binding mode [32]. The Smina [33] software was used to conduct molecular docking analysis. Smina was created by building upon AutoDock Vina [34] to enhance scoring function development and energy minimization. The protein structure was prepared by adding hydrogen atoms, eliminating water molecules and native ligands. The Kollmann charges were assigned to the receptor. To create the compounds, Marvin Sketch [35] was utilized to sketch them and gasteiger charges were assigned by Open Babel [36]. Energy optimization was carried out by Open Babel using the steepest descent algorithm. The active site of the enzyme was then analyzed using Smina to predict the interaction and binding mode of the ligands. To evaluate the computational docking approach, the degree of accuracy was measured using the root mean square deviation (RMSD) value of the re-docking of the cocrystalized ligand back into the CDK8 active site [37].

### Pharmacophore modeling

Pharmacophore methodologies represent successful branches within Computer-Aided Drug Design (CADD), emerging as crucial tools in hit identification, lead optimization, and the rational design of new drugs. A pharmacophore model is a collection of common steric and electronic features that are essential to ensure that the optimal interactions with a specific biological target happen to trigger (or block) its biological response [38]. Various chemical properties, such as Hydrogen Bond Donor (HBD) or Hydrogen Bond Acceptors (HBA), aromatic, cationic, etc., are utilized to characterize interaction patterns [39]. These patterns can be employed to assess the similarity between small molecules within a library and identify the key features contributing to biological activity. Due to the structural diversity of the 12 active compounds, we initially employed ligand-based pharmacophore modeling to identify common features before applying the structure-based pharmacophore modeling stage. We took into account the structural diversity and selected the common feature with the highest score. This information provides valuable insights into our selection of the optimal pharmacophore features in structure-based pharmacophore models. Then we developed a more accurate model using structure-based pharmacophore modeling, which was based on the most active compound (compound 11). We utilized the PharmaGist [40] web server to extract 3D pharmacophores for the 12 active compounds in Table 1. This web server is cost-free and identifies pharmacophores, which refer to the specific spatial arrangement of features that facilitate a molecule’s interaction with a particular target receptor. Multiple flexible alignment of the inhibitors was conducted to identify the highest-scoring pharmacophores. Several common feature models were produced, including those with 5 and 6 features present in all input compounds or only a subset of them. The best model, with a score of 29.047 and five features, including three aromatic and two hydrogen bond acceptors, had the most potent compound 11 as the key molecule (Additional file 1: Fig. S1). In order to select the best feature in Pharmit, we took into account our insights from the output of the PharmaGist server. Pharmit is an online interactive server that allows for the virtual screening of various compound databases using structure-based pharmacophore models and molecular shapes. To validate the models, a set of 100 CDK8 inhibitors with IC50 values less than 1 µM was collected from the literature, along with 5000 decoys suggested by the DUDe server, as a validation dataset. The validation process involved computing the Enrichment Factor (EF) for a search conducted automatically by Pharmit. The enrichment factor is calculated as (Ha/Ht)/(A/D), where D represents the total number of compounds in the database, A represents the total number of active compounds in the database, Ht represents the number of compounds discovered using a particular virtual screening approach, and Ha represents the number of active compounds among Ht [40].

### Molecular dynamics simulation

The GROMACS package version 2019.1 was employed for molecular dynamics simulation (MD) on a GPU Linux server [41]. To conduct MD simulations at 300 K and pH 7, the Amber99sb force field was utilized [42]. The topology was prepared by adding AM1 partial charges using Chimera software [43]. A solute box was defined around the solute, with TIP3P water types used to fill it [44]. Sodium chloride was added to the system at a concentration of 0.15 mol/L to neutralize it. The system’s energy was optimized using a steepest descent algorithm over a 100 ps run. During a 500 ps NVT period, the atom positions of the macromolecule and ligand were restrained using a force constant of 1000 kJ mol^−1^ nm^−2^. V-rescale thermostats were used to maintain the temperature at 300 K during the NVT stage. The system’s pressure was stabilized over the course of a 500 ps equilibration step during the NPT step. The production molecular dynamics (MD) simulation was conducted over a period of 100 ns for model-1, D173A, D189N, T196A, and T196D structures under a well-adjusted system with the 300 K and 1 Bar. The particle-mesh Ewald (PME) algorithm [45] was used to calculate the long-range electrostatic contributions, while the LINCS constraint algorithm was employed to restrict the lengths of covalent bonds, which was three to four times faster than the SHAKE algorithm [46]. At the end of the MD run, the complex was re-centered by returning the protein to the center of the box, and the trajectory was adjusted for periodic boundary conditions. Comparative analysis of the structural deviation in Model-1 and mutant structures, including root mean square deviation (RMSD) and root mean square fluctuation (RMSF), was computed using GROMACS-associated utility packages. The RMSD values were obtained over the entire run to align the protein backbone atoms of each snapshot with the first frame as a reference for determining the equilibrium time range. Additionally, the trajectory was analyzed during the equilibrium time range using the gromos method for cluster analysis [47].

### Virtual screening workflow

Virtual screening is a computational technique used in drug discovery and development to identify potential drug candidates through in-silico (computer-based) screening of large libraries of small molecules [48]. Three stages of virtual screening included structure-based pharmacophore modeling, the identification of drug-like molecules, and molecular docking were done. The Pharmit webserver [49] was used to conduct virtual screening on four databases (CHEMBEL, ZINC, MCULE, and MolPort) in two stages: structure based pharmacophore modeling and the identification of drug-like molecules for model-1 and mutant structures. As mentioned earlier in “Pharmacophore modeling” section, to identify the best features in Pharmit, we considered insights from the PharmaGist server output. Initially, compound 11 was uploaded, followed by the upload of the PDB code to generate the pharmacophore model using Pharmit. Many features generated by PharmaGist were replicated in Pharmit. Subsequently, drug-like molecule properties were selected. These features include molecular weight, log P, topological polar surface area, the number of rotatable bonds, the number of aromatic groups, the number of hydrogen bond acceptors, and the number of hydrogen bond donors. Hits retrieved were additionally refined using molecular docking simulation. The binding modes of inhibitors, along with the critical molecular interactions inside CDK8’s active site with model-1 protein, were investigated using the Smina molecular docking package. Visualization and interaction analyses were performed using the Discovery Studio 2021 Client.

### ADMET study

ADMET is an acronym that stands for Absorption, Distribution, Metabolism, Excretion, and Toxicity, which are all critical factors that affect the pharmacokinetics and pharmacodynamics of a drug [50]. These factors refer to how well a drug is absorbed into the bloodstream, how it reaches its target sites, how the body metabolizes it, how it is eliminated from the body, and how harmful it may be to the body. Understanding the ADMET profile of a drug is crucial in drug discovery and development because it can affect how effective and safe the drug is [51]. By considering these factors, researchers can design drugs that are more effective and less likely to cause harmful side effects. The SwissADME server [52] and the DataWarrior [53] software were used to compute various measures, including the bioavailability score, gastrointestinal absorption, logKp for skin permeation, and toxicity. Also, we employed the web-based tool ProTox-II to forecast the Acute toxicity. Acute toxicity describes the harmful effects of a substance resulting from either a single exposure or multiple exposures in a short period of time (e.g., less than 24 h), with known oral LD50 values measured in rodents. The oral toxicity model includes six different toxicity classes based on the severity of their effect as follows: Class I: fatal if swallowed (LD50 ≤ 5); Class II: highly toxic if swallowed (5 < LD50 ≤ 50); Class III: toxic if swallowed (50 < LD50 ≤ 300); Class IV: harmful if swallowed (300 < LD50 ≤ 2000); Class V: may be harmful if swallowed (2000 < LD50 ≤ 5000); and Class VI: non-toxic (LD50 > 5000) [54].

## Results

### Molecular dynamic analysis

**Fig. 2:**
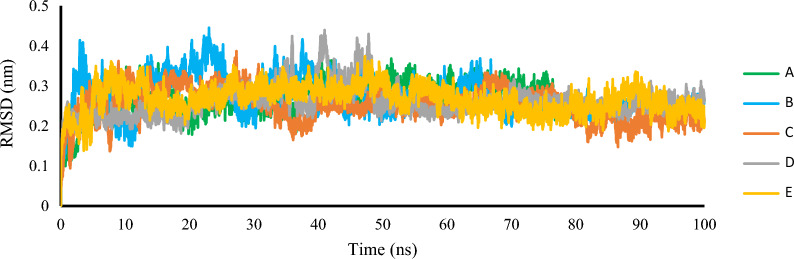
RMSD of **A** model-1 **B** D173A **C** D189N **D** T196A **E** T196D mutations for the backbone atoms in 100 ns MD simulation

The theoretical structure of human CDK8, which included the full length of the A-loop, was generated using homology modeling. The approach involved utilizing an experimentally solved structure (PDB code: 3RGF) as a template to build the structure, resulting in a model called model-1. This particular structure, model-1, was then used in a structure-based method for virtual screening. MD simulation was conducted for the model-1 homology model and the measured RMSD values were used to assess the conformational stability of the model. We aligned the backbone atoms of each frame with those of the first frame and measured RMSD values against time throughout the simulation. The RMSD profile indicated that model-1 achieved equilibrium after 40 ns, as shown in Fig. 2.

**Fig. 3:**
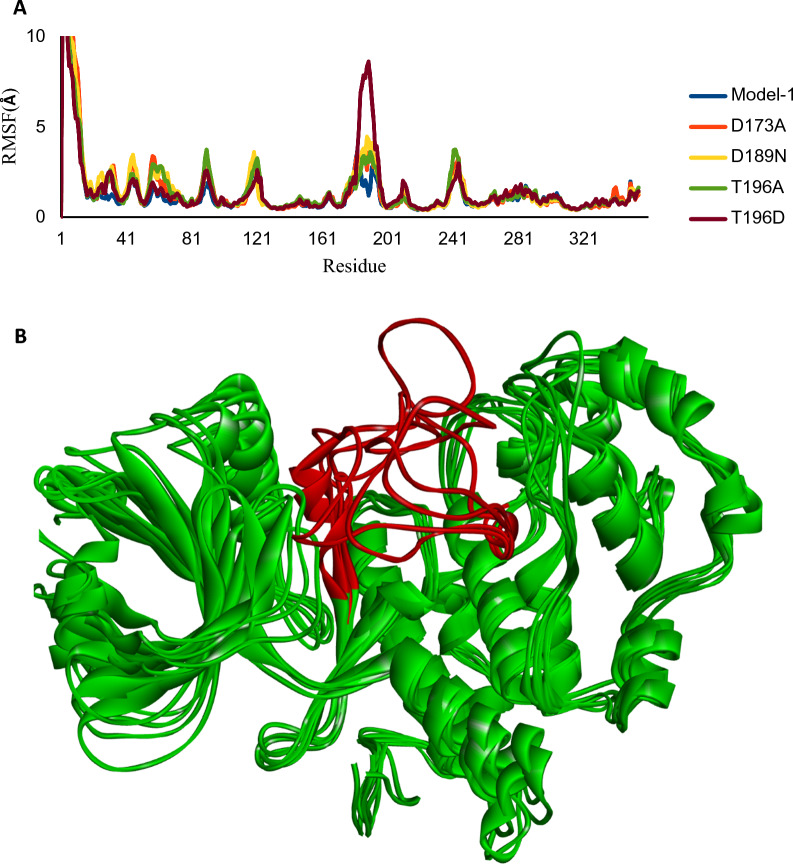
**A** RMSF of the backbone atoms for Model-1 and CDK8 mutants (D173A, D189N, T196A, and T196D). **B** Simulated Model-1 and mutated structures after molecular dynamics simulation. The red highlights represent regions numbered 170-200 where mutations occurred

To identify potential new candidates for mutant structures, we utilized virtual screening filters on extensive libraries. To facilitate this process, we generated the structures of CDK8 with D173A, D189N, T196A, and T196D mutations. In order to evaluate the stability of mutated molecular structures and obtain accurate insights, we carried out simulations using molecular dynamics (MD) for a duration of 100 ns. We verified the stability of all the systems by tracking their Root Mean Square Deviation (RMSD) over time (as shown in Fig. 2). The RMSD results demonstrate that each system achieved a steady, stable state during 100 ns of simulation. Root mean square fluctuation (RMSF) is used to measure the flexibility of each residue and how much the residue moves or fluctuates over a simulation period. The RMSF of the carbon alpha atoms of each residue of model-1 and mutants (D173A, D189N, T196A, and T196D) of CDK8 is calculated in order to analyze the flexibility of backbone structure, which is shown in Fig. 3. The larger RMSF value shows more flexible, whereas low RMSF value shows limited movements during simulation in relation to its average position. It is observed that the backbone atoms of mutant proteins exhibit higher flexibility compared to the Model-1 type. Specifically, the T196D mutant demonstrates increased motion in the residues 170–200 compared to the other trajectories. Model-1 and all mutation structures were aligned for a more consistent analysis. As depicted in Fig. 3B, the entire protein structures are aligned with Model-1, except for regions 170–200 highlighted in red where mutations occurred. This confirms the fluctuations represented in Fig. 3A.

After analyzing the protein structures through molecular dynamics (MD), methods such as molecular docking and pharmacophore screening, which rely on the protein’s structural characteristics, were employed for virtual screening.

### Pharmacophore modeling analysis

**Fig. 4:**
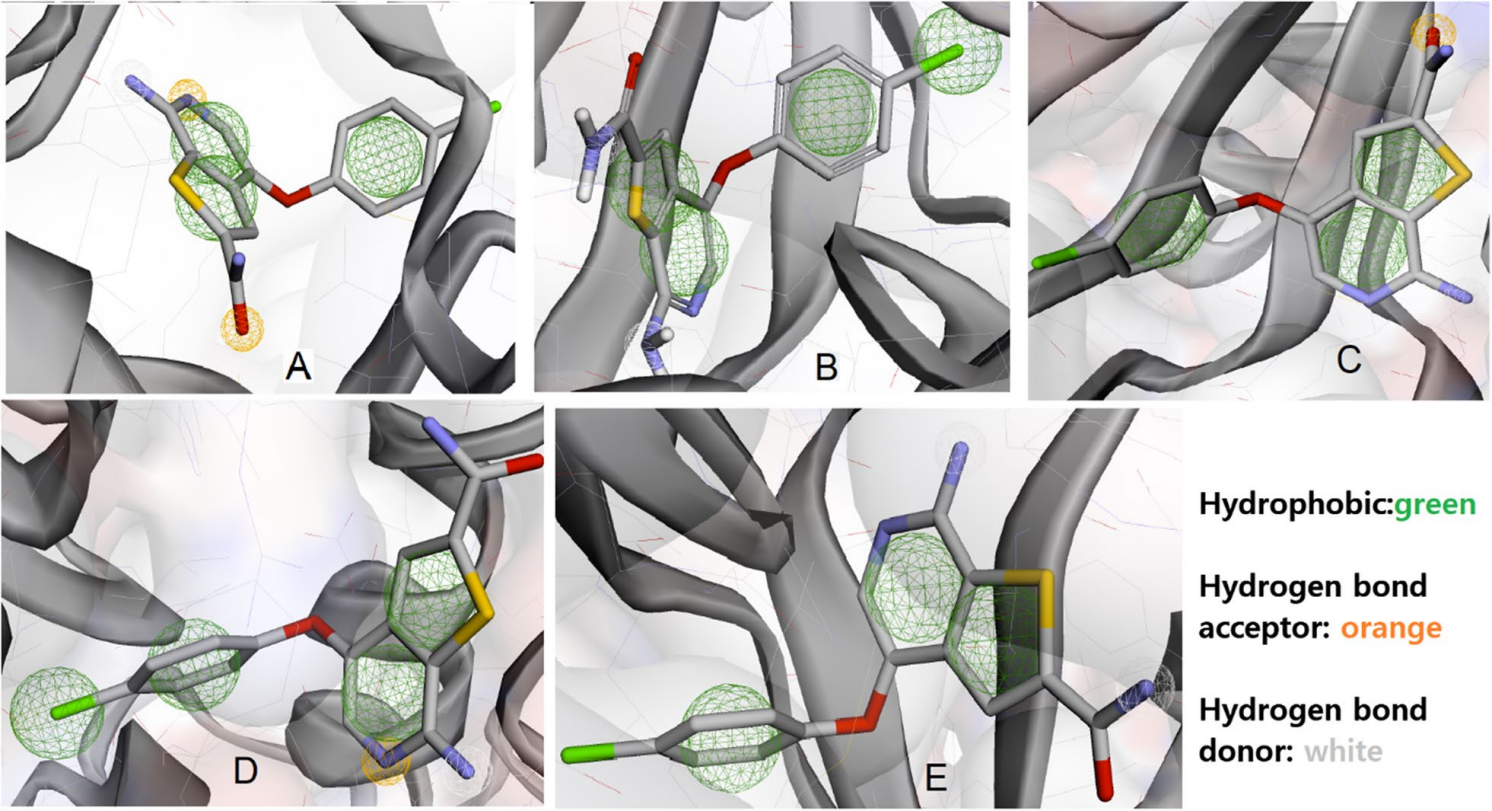
Pharmacophore models of compound 11 with **A** model-1 **B** D173A **C** D189N **D** T196A **E** T196D

Utilizing our insights from PharmaGist (a ligand-based pharmacophore model), we carefully chose the optimal pharmacophore features in Pharmit (a structure-based pharmacophore model). The models were conducted by emphasizing the key residues involved in active site interactions of the homology model and mutation structures with compound 11 as the most active compound. An Enrichment Factor between 3 and 100 indicates that a specific virtual screening approach saves significant time, costs, and experimentation. The EF values for the models had a range of 6.2 to 18.6, as depicted in Table 2. This range implies that our screening approach may lead to a higher rate of success. Figure 4 illustrates the features of pharmacophore models for compound 11, along with the homology model and mutated protein structures.

**Table 2:** Enrichment factor for pharmacophore models of each protein with compound 11

Protein’s name	Enrichment factor
Model-1 (homology model)	6.8
D173A	18.6
D189N	16.9
T196A	8.9
T196D	6.2

### Molecular docking and virtual screening analysis

**Fig. 5:**
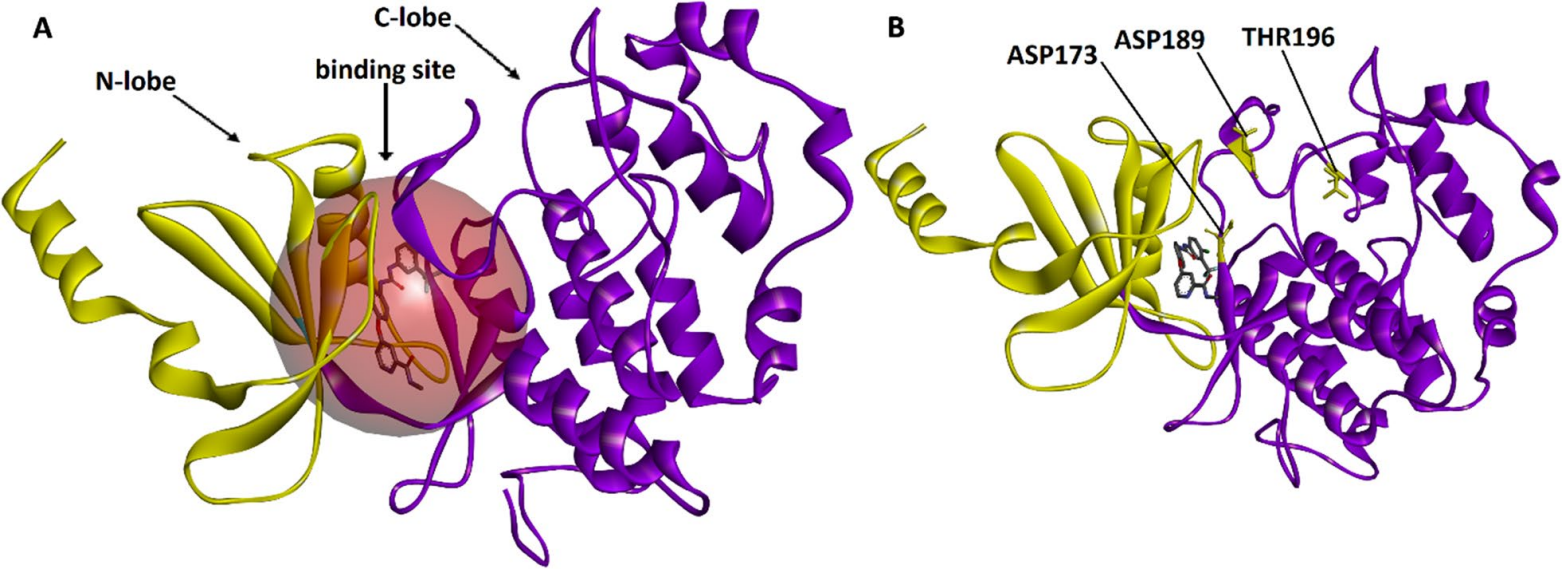
Overview of the structure of CDK8 receptor (**A**) and mutations position (**B**)

The CDK8 protein is composed of several structural elements, such as the N-lobe (N-terminal lobe, residues 1–96) and the C-lobe (C-terminal lobe, residues 97–353). Generally, the ATP-binding site of the kinase is located between the N-lobe and C-lobe. The N-lobe is characterized by a β-sheet, while the C-lobe includes α-helices and loops [55]. The hypothetical CDK8 structure is shown in Fig. 5A. The regions in the CDK structure where mutations were introduced, along with their positions relative to the binding site, were illustrated in Fig. 5B.

**Fig. 6:**
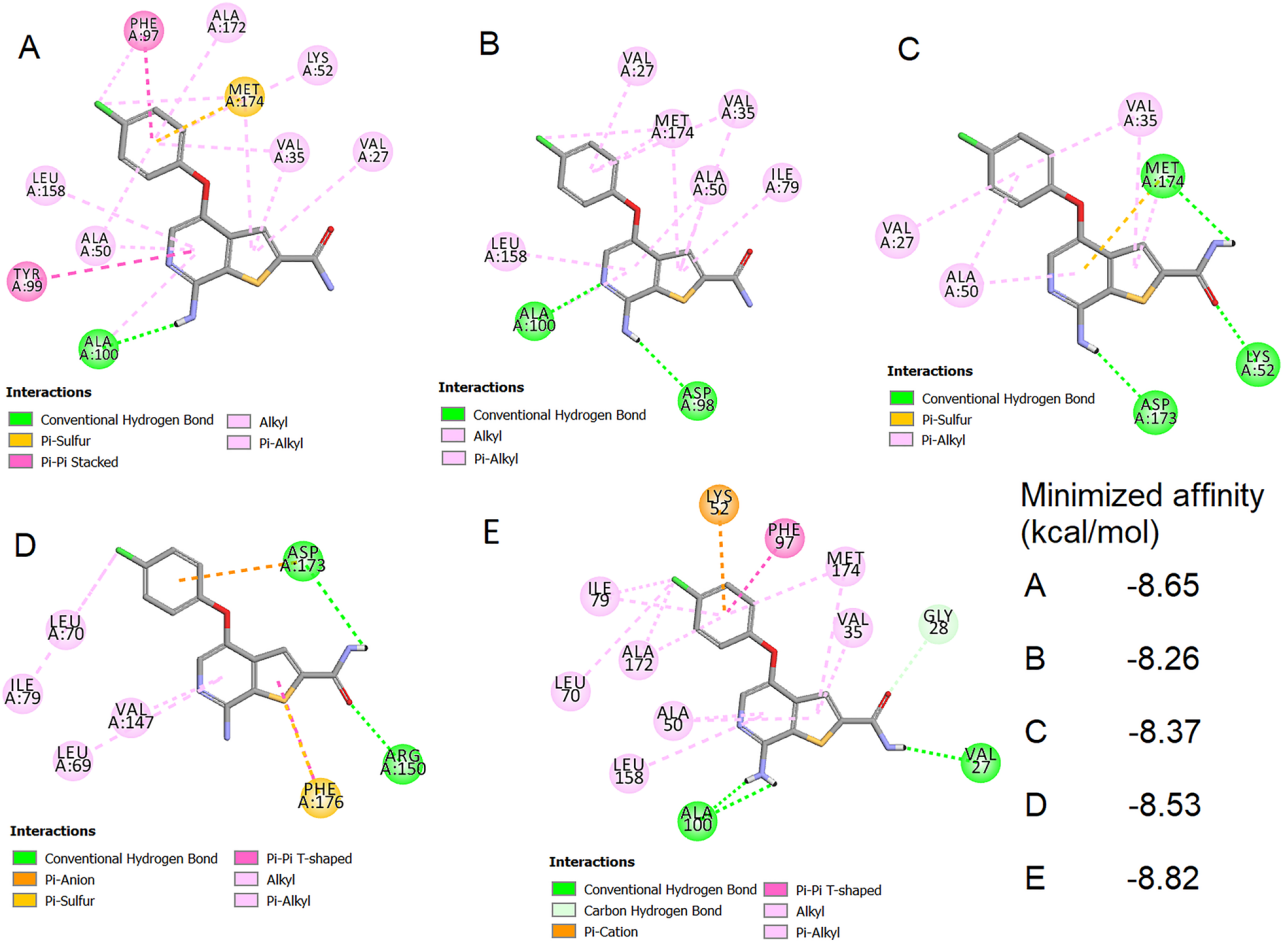
Molecular docking analysis of the interaction of compound 11 with **A** model-1 **B** D173A **C** D189N **D** T196A **E** T196D. Images were created by Discovery Studio 2021 Client

Using Smina docking, a molecular docking analysis was conducted to assess the capacity of compound 11 to interact with the model-1 and mutation structures. When the native ligand was placed back into the model-1 active site, the resulting RMSD value was 1.6 Å, which falls below the maximum acceptable value of 2 Å (Additional file 1: Fig. S2). Figure 6 presents the minimized affinity values and interactions of compound 11 with all protein structures in two dimensional. The three-dimensional structures of all interactions were depicted, and distance of each interaction pair were shown in Additional file 1: Fig. S3.

Among the databases supported by Pharmit, we opted to screen the ZINC, CHEMBL32, MCULE, and MolPort databases due to the availability of purchasable compounds for virtual screening. Then, the filter was established using typical molecular characteristics for recognizing drug-like molecules. These features comprise molecular weight, log P (a measurement of lipophilicity), topological polar surface area (a sign of the compound’s ability to penetrate cell membranes), the number of rotatable bonds, the number of aromatic groups, the number of hydrogen bond acceptors, and the number of hydrogen bond donors. Open Babel is utilized to precompute these features. Smina was utilized in the third screen to confirm that the search into the binding site of the homology model and mutation structures produced all hits. The compounds were then sorted based on their docking score values, and only the ones with scores higher than compound 11 were included in the list. Table 3 presents the hits obtained from virtual screening procedures across all four databases for each protein. This table illustrates that during the second screening step, a certain number of compounds were subjected to docking simulations using different protein structures. Specifically, 50, 154, 405, 366, and 356 compounds were docked into the model-1, D173A, D189N, T196A, and T196D structures, respectively. After three stages of the virtual screening mean structure-based pharmacophore modeling, the identification of drug-like molecules, and molecular docking, a total of 13, 11, 11, 15, and 12 relevant results were selected for model-1, D173A, D189N, T196A, and T196D structures, correspondingly. The structures and molecular docking minimized affinity values of these hits were shown in Additional file 1: Table S1 and the affinity score with the lowest value (indicating the most negative) each protein was emphasized in bold.

**Table 3:** Hits obtained from virtual screening procedures

Database	Number of compounds	Pharmacophore model filter	Drug-like molecules filter	Molecular docking filter
Model-1 (homology model)				
CHEMBL32	2,186,411	247	13	0
MCULE	45,257,086	204	16	8
MolPort	4,843,718	345	4	0
ZINC	12,921,916	561	17	5
Total	65,209,131	1357	50	13
D173A				
CHEMBL32	2,186,411	748	13	1
MCULE	45,257,086	281	31	5
MolPort	4,843,718	579	78	4
ZINC	12,921,916	611	32	1
Total	65,209,131	2219	154	11
D189N				
CHEMBL32	2,186,411	258	69	1
MCULE	45,257,086	330	109	5
MolPort	4,843,718	303	101	5
ZINC	12,921,916	337	126	0
Total	65,209,131	1228	405	11
T196A				
CHEMBL32	2,186,411	1290	108	10
MCULE	45,257,086	1447	81	1
MolPort	4,843,718	1392	86	1
ZINC	12,921,916	1581	91	3
Total	65,209,131	5710	366	15
T196D				
CHEMBL32	2,186,411	1006	45	3
MCULE	45,257,086	526	69	4
MolPort	4,843,718	1306	127	3
ZINC	12,921,916	1251	115	2
Total	65,209,131	4089	356	12

**Fig. 7:**
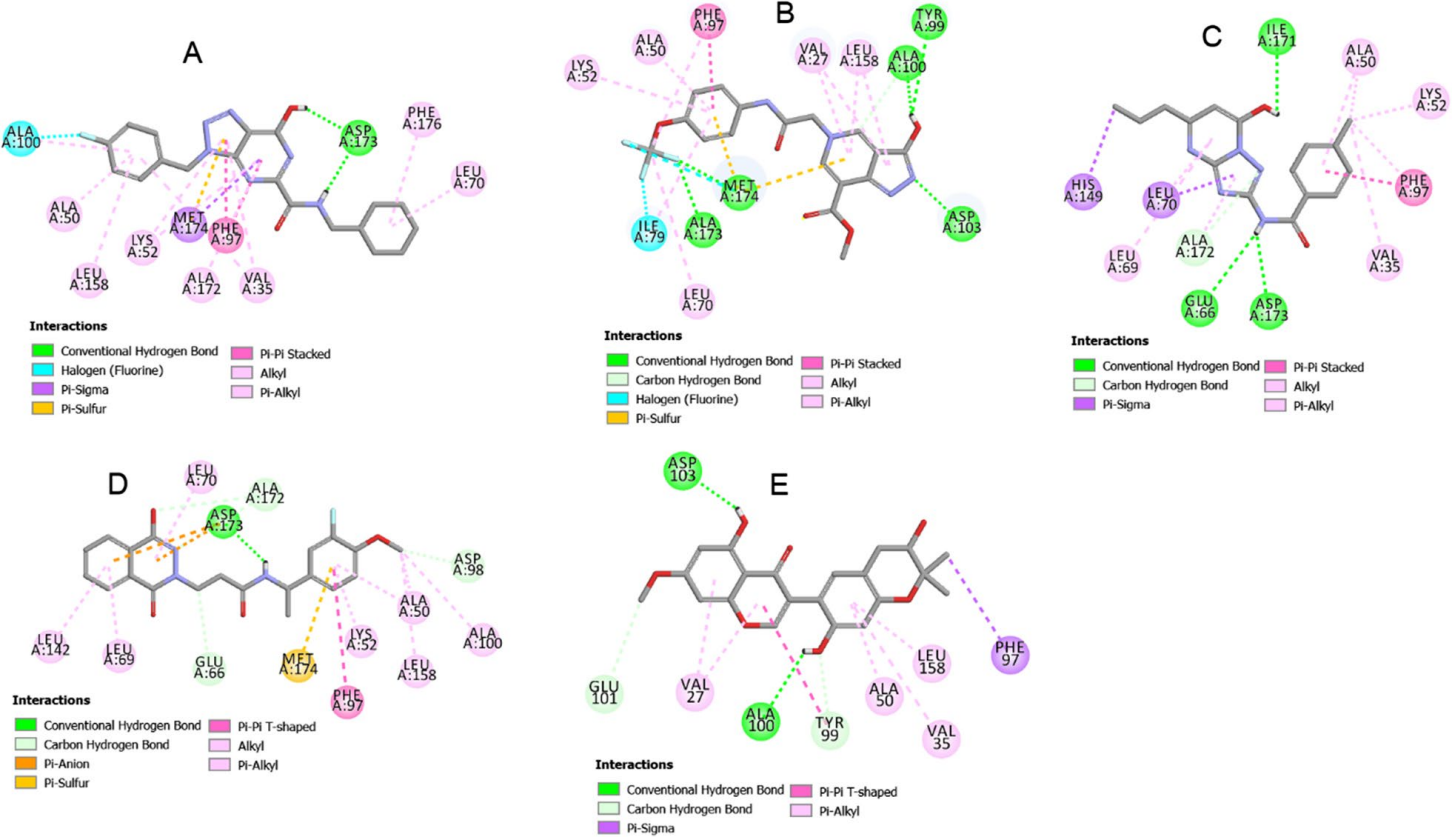
Molecular docking analysis of the interaction of **A** compound M1 with model-1 **B** compound N7 with D173A **C** compound P7 with D189N **D** compound Q13 with T196A **E** compound R1 with T196D. Images were created by Discovery Studio 2021 Client

Figure 7 displays the docking interaction patterns between the best hit compounds (M1, N7, P7, Q13, and R1) respectively in term of energy (Additional file 1: Table S1), with model-1, D173A, D189N, T196A, and T196D structures. The three-dimensional structures of all interactions were depicted, and distance of each interaction pair were shown in Additional file 1: Fig. S4. These patterns were then compared to the interaction of each protein with compound 11, and the shared key residues were identified and listed in Table 4. The identification of shared key residues can provide insights into the structural and functional characteristics of the protein, as well as the potential binding modes and selectivity of the compounds. Compound 11 is an organic compound called a diarylether. On the other hand, compounds M1 and P7 fall into the category of triazolopyrimidines, which are aromatic compounds with a triazole ring fused to a pyrimidine ring. Meanwhile, compound N7 is a pyrazolopyridine, characterized by a structure containing a pyrazole fused to a pyridine. Compound Q13 is classified as a phthalazinone, featuring a phthalazine with a ketone group. Lastly, compound R1 is a pyranoisoflavonoid, an isoflavonoid with a pyran ring fused to either the A, B, or C ring of its structure. These newly screened molecules have a distinct scaffold and differ from compound 11. This information can be useful in drug discovery and development, as it can guide the design and optimization of compounds with improved potency and selectivity.

**Table 4:** Interaction of each protein with compound 11, hit compounds from virtual screening, and shared key residues

Complex	Interactions	Shared key residues
Model-1/compound 11	VAL27, VAL35, ALA50, LYS52, PHE97, TYR99, ALA100, LEU158, ALA172, MET174	VAL35, ALA50, LYS52, PHE97, ALA100, LEU158, ALA172, MET174
Model-1/M1	VAL35, ALA50, LYS52, LEU70, PHE97, ALA100, LEU158, ALA172, ASP173, MET174, PHE176	
D173A/compound 11	VAL27, VAL35, ALA50, ILE79, ASP98, ALA100, LEU158, MET174	VAL27, ALA50, ILE79, ALA100, LEU158, MET174
D173A/N7	VAL27, ALA50, LYS52, LEU70, ILE79, PHE97, TYR99, ALA100, ASP103, LEU158, ALA173, MET174	
D189N/compound 11	VAL27, VAL35, ALA50, LYS52, ASP173, MET174	VAL35, ALA50, LYS52, ASP173
D189N/P7	VAL35, ALA50, LYS52, GLU66, LEU69, LEU70, PHE97, HIS149, ILE171, ALA172, ASP173	
T196A/compound 11	LEU69, LEU70, ILE79, VAL147, ARG150, ASP173, PHE176	LEU69, LEU70, ASP173
T196A/Q13	ALA50, LYS52, GLU66, LEU69, LEU70, PHE97, ASP98, ALA100, LEU142, LEU158, ALA172, ASP173, MET174	
T196D/compound 11	VAL27, GLY28, VAL35, ALA50, LYS52, LEU70, ILE79, PHE97, ALA100, LEU158, ALA172, MET174	VAL27, VAL35, ALA50, PHE97, ALA100, LEU158
T196D/R1	VAL27, VAL35, ALA50, PHE97, TYR99, ALA100, GLU101, ASP103, LEU158	

### ADMET properties

The SwissADME server and DataWarrior was employed to predict the pharmacokinetic characteristics and toxicity properties for all compounds. During the virtual screening step, Pharmit filters compounds during hit screening based on drug-like properties including number of rotatable bonds, molecular weight, logP, topological polar surface area, number of HBAs, number of HBDs, and number of aromatic groups. The specified ranges for these properties are: MW ≤ 500, rotatable bonds ≤ 10, logP ≤ 5, PSA ≤ 140 Å2, aromatic groups ≤ 5, 2 ≤ HBA ≤ 7, and 2 ≤ HBD ≤ 7. The Abbott bioavailability score is used to assess drug-likeness, with a score of 0.55 indicating that the best-predicted hits from virtual screening passed the rule-of-five. Skin permeability, logKp was also calculated and fell within the standard range of − 1 to − 8 for 95% of drugs [49]. Bioavailability is regulated by several factors, but the most important determinant is gastrointestinal absorption [50]. In this study, the reported hits were found to have high gastrointestinal absorption. To ensure the safety of these hits, their toxicity risk was evaluated for potential mutagenic, tumorigenic, irritant, and reproductive effects. Reproductive toxicity may cause alterations to the male and female reproductive systems, while irritant toxicity can cause reversible damage to the skin or other organs. According to the predictions of acute toxicity or LD_50_ value, most hits (except N4, N6, Q6, R3, R4, and R10) are classified as Class 4. Therefore, they are considered “harmful if swallowed” (300 < LD_50_ ≤ 2000). Based on its toxicological qualities, they are not considered to pose a risk for protein toxicity. A majority of the virtual screening hits exhibited satisfactory molecular properties, as shown in Additional file 1: Table S2.

## Conclusion

In this study, we employed 12 known CDK8 inhibitors to perform a comprehensive search of four databases (CHEMBEL, ZINC, MCULE, and MolPort) containing 65,209,131 molecules. Our objective was to identify potent inhibitors targeting the CDK8 protein and its single mutations, namely D189N, D173A, T196A, and T196D. By filtering molecules based on structure-based pharmacophore modeling, drug-like molecule analysis, and molecular docking, we selected a total of 13, 11, 11, 15, and 12 compounds as potential inhibitors for the model-1, D173A, D189N, T196A, and T196D structures, respectively. Notably, the compounds M1 (MCULE-7964427553, ZINC000035315561), N7 (MolPort-042-647-630, MCULE-4160838791), P7 (MolPort-007-690-145, ChemDiv-D205-0374, MCULE-4441768704, ZINC09202982), Q13 (ZINC000007040960), and R1 (CHEMBL1077871) exhibited the most favorable energy interactions with their respective target structures, suggesting their potential as effective inhibitors. However, it is important to conduct experimental assays to validate these identified compounds as hit molecules and further optimize their potency as inhibitors.

### Supplementary Information

**Additional file 1: Table S1.** Hits retrieved from the virtual screening alongside their minimized affinity values. **Table S2.** The projected ADMET characteristics for the identified hits. **Figure S1.** Ligand-based pharmacophore model aligned on compound 11. **Figure S2.** Superimposing the crystallized ligands (yellow) and redocked ligands (magenta) at CDK8 with the 3RGF PDB code to validate the docking protocol. **Figure S3.** The three-dimensional structures of molecular docking of compound 11 with (A) model-1 (B) D173A (C) D189N (D) T196A (E) T196D. Images were created by Discovery Studio 2021 Client. **Figure S4.** The three-dimensional structures of molecular docking of (A) compound M1 with model-1 (B) compound N7 with D173A (C) compound P7 with D189N (D) compound Q13 with T196A (E) compound R1 with T196D. Images were created by Discovery Studio 2021 Client.

## Data Availability

All data generated or analyzed during this study are included in this published article and its Additional file.
