# Supplementary material for: Hit discovery of potential CDK8 inhibitors and analysis of amino acid mutations for cancer therapy through computer-aided drug discovery
Source: BMC Chem. 2024 Apr 13;18(1):73. doi: 10.1186/s13065-024-01175-6 (PMC11016228; doi:10.1186/s13065-024-01175-6)
Supplement: Supplementary file 1 — Additional file 1: Table S1. Hits retrieved from the virtual screening alongside their minimized affinity values. Table S2. The projected ADMET characteristics for the identified hits. Figure S1. Ligand-based pharmacophore model aligned on compound 11. Figure S2. Superimposing the crystallized ligands (yellow) and redocked ligands (magenta) at CDK8 with the 3RGF PDB code to validate the docking protocol. Figure S3. The three-dimensional structures of molecular docking of compound 11 with (A) model-1 (B) D173A (C) D189N (D) T196A (E) T196D. Images were created by Discovery Studio 2021 Client. Figure S4. The three-dimensional structures of molecular docking of (A) compound M1 with model-1 (B) compound N7 with D173A (C) compound P7 with D189N (D) compound Q13 with T196A (E) compound R1 with T196D. Images were created by Discovery Studio 2021 Client. [file 13065_2024_1175_MOESM1_ESM.docx]

**Hit discovery of potential CDK8 inhibitors and analysis of amino acid mutations for cancer therapy through computer-aided drug discovery**

Raziye Aghahasani^1^, Fereshteh Shiri^2,*^, [Hossein Kamaladiny](https://cropscience.uoz.ac.ir/?_action=article&au=1009706&_au=Hossein++Kamaladiny&lang=en)^1^, [Fatemeh Haddadi](https://cropscience.uoz.ac.ir/?_action=article&au=1142727&_au=Fatemeh++Haddadi&lang=en)^1^ & Somayeh Pirhadi^3^

*^1^Department of Biology, University of Zabol, Zabol, Iran*

*^2^Department of Chemistry, University of Zabol, Zabol, Iran*

*^3^Medicinal and Natural Products Chemistry Research Center, Shiraz University of Medical Sciences, Shiraz, Iran*

E-mail addresses:

F. Shiri^*^: [fereshteh.shiri@gmail.com](mailto:fereshteh.shiri@gmail.com) & Fereshteh.shiri@uoz.ac.ir

Tel./fax: +98 5431232186

| Table S1 Hits retrieved from the virtual screening alongside their minimized affinity values. | | | |
| --- | --- | --- | --- |
| No. | Hit name in database | Structure | Minimized affinity (kcal/mol) |
| Model-1(Homology model) | | | |
| **M1** | **MCULE-7964427553 ZINC000035315561** | **** | **-9.92** |
| M2 | MCULE-6662376328 ChemDiv-8018-6036 MolPort-002-033-313 ZINC000012374481 |  | -9.84 |
| M3 | MCULE-6692963415 ZINC000012113107 |  | -9.55 |
| M4 | MCULE-3195198385 ZINC000035315573 |  | -9.47 |
| M5 | MCULE-9656861453 ZINC000035315571 |  | -9.38 |
| M6 | MCULE-2870256641 ZINC000035315566 |  | -9.17 |
| M7 | MCULE-3858343759 ZINC000035315572 |  | -9.09 |
| M8 | MCULE-7891382609 ZINC000005059028 |  | -9.05 |
| M9 | ZINC000035315565 MCULE-3486960696 |  | -9.91 |
| M10 | ZINC000012113107 |  | -9.52 |
| M11 | ZINC000035315575 MCULE-2479966564 |  | -9.40 |
| M12 | ZINC000035310822 |  | -9.35 |
| M13 | ZINC000035315566 |  | -9.17 |
| D173A | | | |
| N1 | CHEMBL2098347 PubChem-22320081 |  | -9.53 |
| N2 | MCULE-5170347328 MolPort-042-647-627 |  | -10.21 |
| N3 | MCULE-9479627207 LN00811678 MolPort-005-976-364 |  | -9.99 |
| N4 | MCULE-1114858979 MolPort-042-647-626 |  | -9.91 |
| N5 | MCULE-9235183449 |  | -9.36 |
| N6 | MCULE-1450951927 CSC084804122 MolPort-046-811-641 |  | -9.23 |
| **N7** | **MolPort-042-647-630 MCULE-4160838791** | **** | **-10.46** |
| N8 | MolPort-000-766-940 |  | -9.98 |
| N9 | MolPort-002-799-204 MCULE-1176825268 PubChem-1045373 ZINC000000719457 ZINC00719457 |  | -9.51 |
| N10 | MolPort-042-647-664 MCULE-9173786203 |  | -9.41 |
| N11 | ZINC000565801159 |  | -9.85 |
| D189N | | | |
| P1 | CHEMBL2426786 PubChem-46240836 |  | -9.34 |
| P2 | MCULE-8973955812 ChemDiv-D205-0375 MolPort-002-128-201 ZINC000009202987 |  | -10.39 |
| P3 | MCULE-3368514902 ChemDiv-D205-0386 MolPort-007-690-153 ZINC000009203013 ZINC09203013 |  | -10.22 |
| P4 | MCULE-9528415409 MolPort-046-423-561 ZINC001547205783 |  | -9.78 |
| P5 | MCULE-7724163420 ChemDiv-D205-0385 MolPort-007-690-152 ZINC000009203006 ZINC09203006 |  | -9.72 |
| P6 | MCULE-5485663814 ZINC000019210723 |  | -9.52 |
| **P7** | **MolPort-007-690-145 ChemDiv-D205-0374 MCULE-4441768704 ZINC09202982** | **** | **-10.85** |
| P8 | MolPort-002-306-017 ChemDiv-D205-0373 MCULE-1623394044 ZINC000009202978 |  | -10.20 |
| P9 | MolPort-007-690-147 ChemDiv-D205-0379 MCULE-8661654422 ZINC000021171411 |  | -10.09 |
| P10 | MolPort-046-853-858 |  | -9.67 |
| P11 | MolPort-000-821-108 ChemDiv-D205-0372 MCULE-1972884353 ZINC000008994804 |  | -9.48 |
| T196A | | | |
| Q1 | CHEMBL1723826 |  | -9.47 |
| Q2 | CHEMBL3968050 |  | -9.31 |
| Q3 | CHEMBL3445584 MCULE-6182171294 MCULE-8986078170 MolPort-009-758-744 MolPort-016-588-662 |  | -9.26 |
| Q4 | CHEMBL2315831 PubChem-69480435 |  | -9.18 |
| Q5 | CHEMBL2316124 PubChem-69480289 |  | -9.15 |
| Q6 | CHEMBL2147149 |  | -9.13 |
| Q7 | CHEMBL2316138 |  | -9.10 |
| Q8 | CHEMBL3309907 PubChem-69482441 |  | -9.09 |
| Q9 | CHEMBL3950775 |  | -9.07 |
| Q10 | CHEMBL257269 PubChem-44455012 |  | -9.00 |
| Q11 | MCULE-8925695307 MolPort-030-041-658 ZINC000059446055 |  | -9.32 |
| Q12 | MolPort-027-845-872 |  | -9.05 |
| **Q13** | **ZINC000007040960** | **** | **-9.86** |
| Q14 | ZINC000952984877 |  | -9.68 |
| Q15 | ZINC000041149936 |  | -9.16 |
| T196D | | | |
| **R1** | **CHEMBL1077871** | **** | **-10.14** |
| R2 | CHEMBL389702 PubChem-44422252 |  | -10.07 |
| R3 | CHEMBL4075682 |  | -9.44 |
| R4 | MCULE-9853187458 MolPort-046-515-466 |  | -9.87 |
| R5 | MCULE-9528415409 MolPort-046-423-561 ZINC001547205783 |  | -9.83 |
| R6 | MCULE-1952231667 |  | -9.40 |
| R7 | MCULE-5670972269 MolPort-046-424-092 ZINC001547208795 |  | -9.39 |
| R8 | MolPort-007-830-229 ChemDiv-G199-3196 MCULE-2928562606 PubChem-20942592 ZINC000009641647 |  | -9.39 |
| R9 | MolPort-007-830-185 ChemDiv-G199-2982 MCULE-3863551671 PubChem-46337881 ZINC000021911252 |  | -9.38 |
| R10 | MolPort-046-560-753 MCULE-5261903179 |  | -9.31 |
| R11 | ZINC000118927178 PubChem-99572761 |  | -9.71 |
| R12 | ZINC000118930659 PubChem-99574134 |  | -9.57 |

| Table S2 The projected ADMET characteristics for the identified hits. | | | | | | | |  |
| --- | --- | --- | --- | --- | --- | --- | --- | --- |
| Name | Bioavailability Score | GI absorption | log Kp (cm/s) | Mutagenic | Tumorigenic | Reproductive Effective | Irritant | LD_50_(mg/kg) |
| M1 | 0.55 | High | -6.25 | none | none | none | none | 500 |
| M2 | 0.55 | High | -6.26 | none | none | none | none | 1190 |
| M3 | 0.55 | High | -7.03 | none | none | none | none | 500 |
| M4 | 0.55 | High | -6.73 | none | none | none | none | 348 |
| M5 | 0.55 | High | -6.9 | none | none | none | high | 500 |
| M6 | 0.55 | High | -7.18 | high | none | none | none | 500 |
| M7 | 0.55 | High | -6.9 | none | none | none | none | 500 |
| M8 | 0.55 | High | -6.77 | none | none | none | none | 500 |
| M9 | 0.55 | High | -7.03 | none | none | none | none | 500 |
| M10 | 0.55 | High | -7.03 | none | none | none | none | 500 |
| M11 | 0.55 | High | -6.73 | none | none | none | none | 500 |
| M12 | 0.55 | High | -6.7 | none | none | none | none | 500 |
| M13 | 0.55 | High | -7.18 | high | none | none | none | 500 |
| N1 | 0.55 | High | -7.19 | none | none | none | none | 1000 |
| N2 | 0.55 | High | -7.04 | none | none | high | none | 460 |
| N3 | 0.55 | High | -7.11 | none | none | none | none | 1000 |
| N4 | 0.55 | High | -6.26 | none | none | low | none | 130 |
| N5 | 0.55 | High | -6.95 | none | none | none | none | 2000 |
| N6 | 0.55 | High | -6.51 | none | none | low | high | 130 |
| N7 | 0.55 | High | -7.65 | none | none | none | none | 1000 |
| N8 | 0.55 | High | -6.56 | none | none | none | none | 2000 |
| N9 | 0.55 | High | -7.21 | none | none | none | none | 1800 |
| N10 | 0.55 | High | -7.74 | none | none | none | low | 2000 |
| N11 | 0.55 | High | -7.62 | none | none | none | none | 1000 |
| P1 | 0.55 | High | -7.28 | none | none | none | high | 501 |
| P2 | 0.55 | High | -6.43 | none | none | none | none | 300 |
| P3 | 0.55 | High | -6.15 | none | none | none | none | 300 |
| P4 | 0.55 | High | -7.1 | none | none | none | none | 5000 |
| P5 | 0.55 | High | -6.21 | none | none | none | none | 300 |
| P6 | 0.55 | High | -7.4 | none | none | none | none | 800 |
| P7 | 0.55 | High | -6.21 | none | none | none | none | 300 |
| P8 | 0.55 | High | -6.15 | none | none | none | none | 300 |
| P9 | 0.55 | High | -6.59 | none | none | none | none | 300 |
| P10 | 0.55 | High | -6.67 | none | none | none | none | 1000 |
| P11 | 0.55 | High | -6.4 | none | none | none | none | 300 |
| Q1 | 0.55 | High | -6.87 | none | none | none | none | 900 |
| Q2 | 0.55 | High | -7.31 | high | none | none | none | 1000 |
| Q3 | 0.55 | High | -6.46 | high | high | none | none | 500 |
| Q4 | 0.55 | High | -7.95 | none | none | none | none | 600 |
| Q5 | 0.55 | High | -7.26 | none | none | none | none | 750 |
| Q6 | 0.55 | High | -6.74 | none | none | none | none | 13 |
| Q7 | 0.55 | High | -7.35 | none | none | none | none | 1500 |
| Q8 | 0.55 | High | -7.61 | none | none | none | none | 800 |
| Q9 | 0.55 | High | -7.33 | none | none | none | none | 1200 |
| Q10 | 0.55 | High | -6.8 | none | none | none | none | 850 |
| Q11 | 0.55 | High | -6 | none | high | none | low | 1000 |
| Q12 | 0.55 | High | -6.25 | none | none | none | none | 800 |
| Q13 | 0.55 | High | -7.11 | none | none | none | none | 500 |
| Q14 | 0.55 | High | -7 | none | none | none | none | 300 |
| Q15 | 0.55 | High | -6.46 | high | high | none | none | 500 |
| R1 | 0.55 | High | -6.51 | none | none | high | none | 2875 |
| R2 | 0.55 | High | -7.2 | none | high | none | none | 775 |
| R3 | 0.55 | High | -7.34 | none | none | none | none | 1 |
| R4 | 0.55 | High | -7.39 | none | none | none | none | 200 |
| R5 | 0.55 | High | -7.1 | none | none | none | none | 5000 |
| R6 | 0.55 | High | -7.52 | none | high | none | high | 460 |
| R7 | 0.55 | High | -7.16 | none | none | none | none | 1820 |
| R8 | 0.55 | Low | -7.34 | none | none | none | none | 1000 |
| R9 | 0.55 | High | -7.13 | none | none | none | none | 1000 |
| R10 | 0.55 | High | -8.07 | none | none | none | none | 200 |
| R11 | 0.55 | High | -7.65 | none | none | high | none | 1000 |
| R12 | 0.55 | High | -7.71 | none | none | high | none | 1170 |


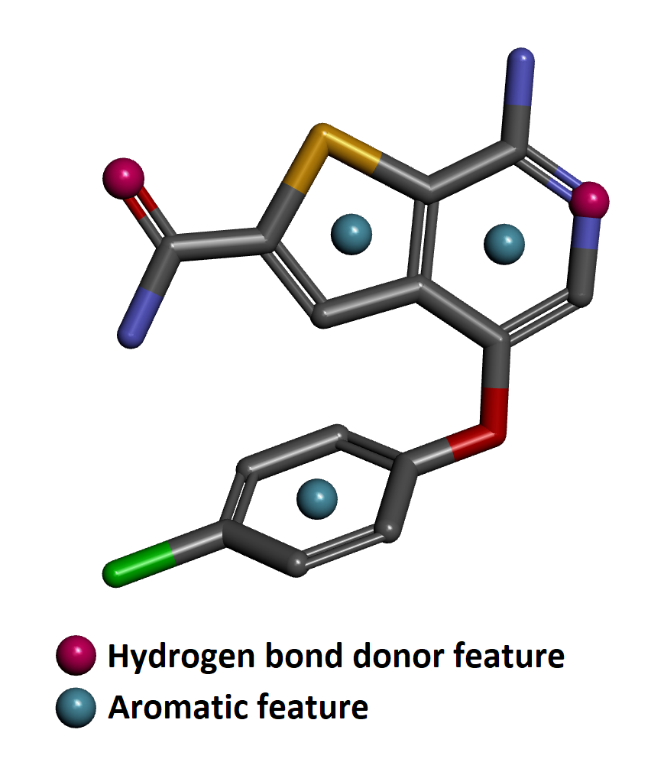


Figure S1 Ligand- based Pharmacophore model aligned on compound 11


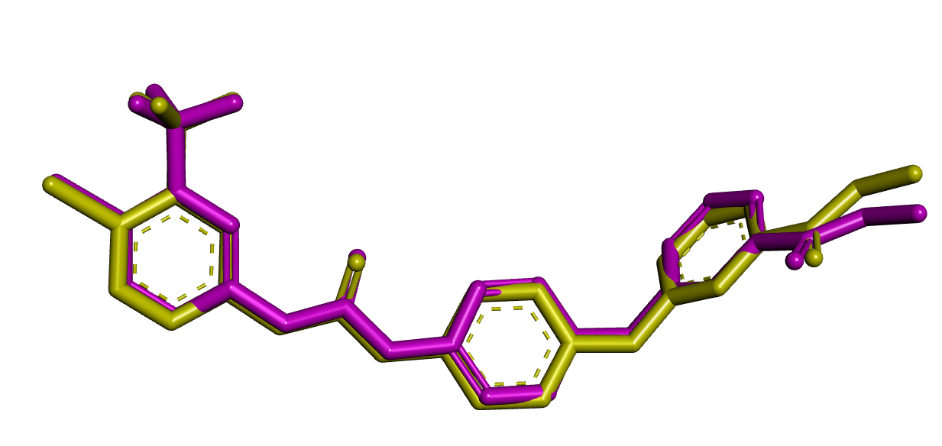


Figure S2 Superimposing the crystallized ligands (yellow) and redocked ligands (magenta) at CDK8 with the 3RGF PDB code to validate the docking protocol.


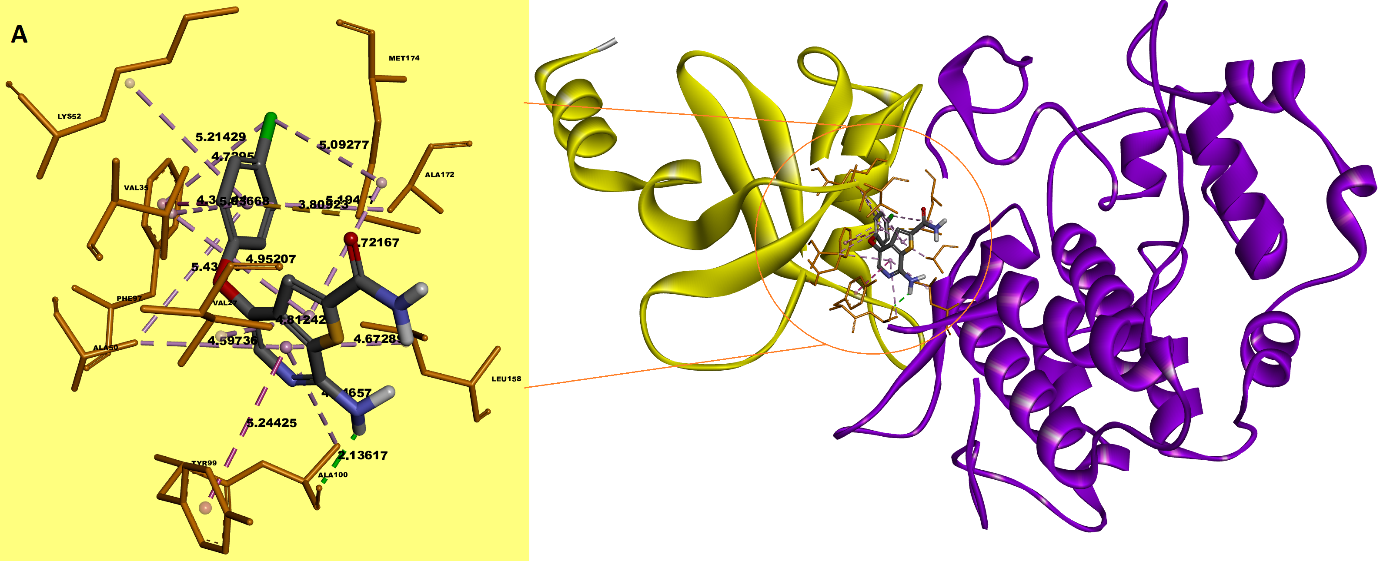


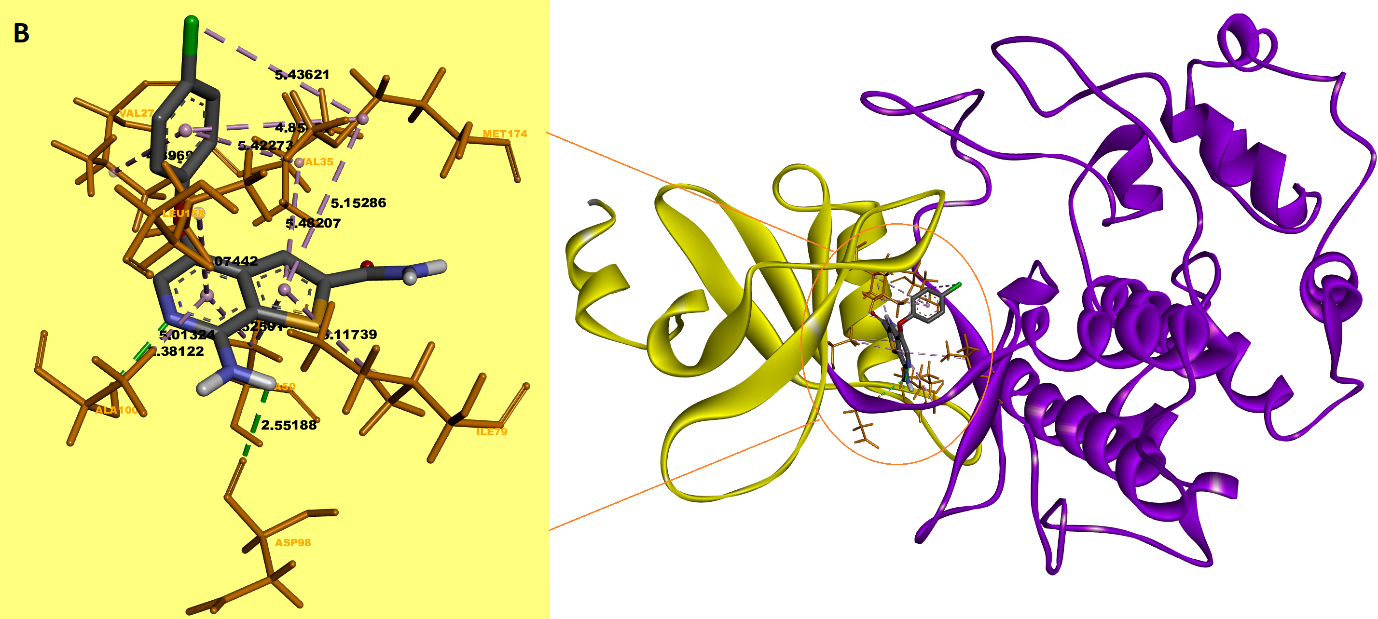


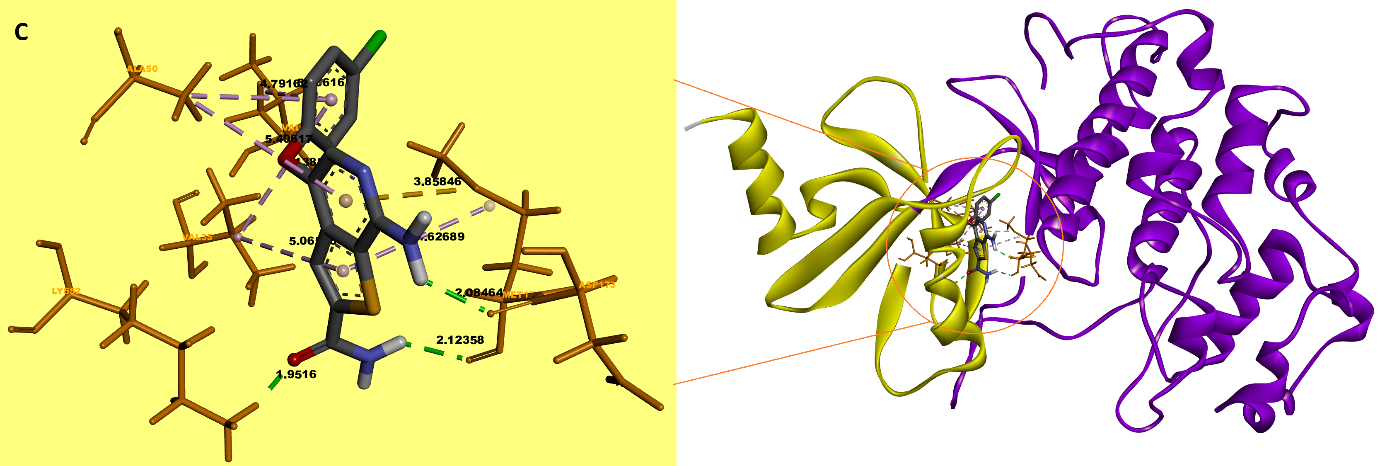


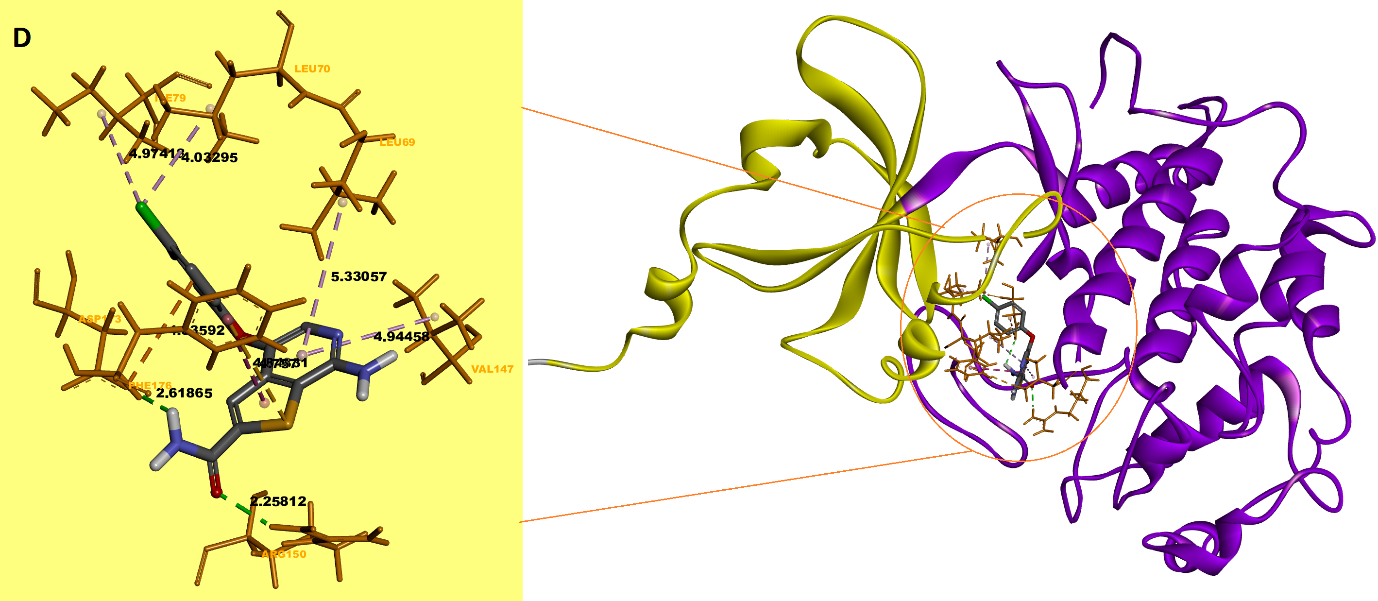


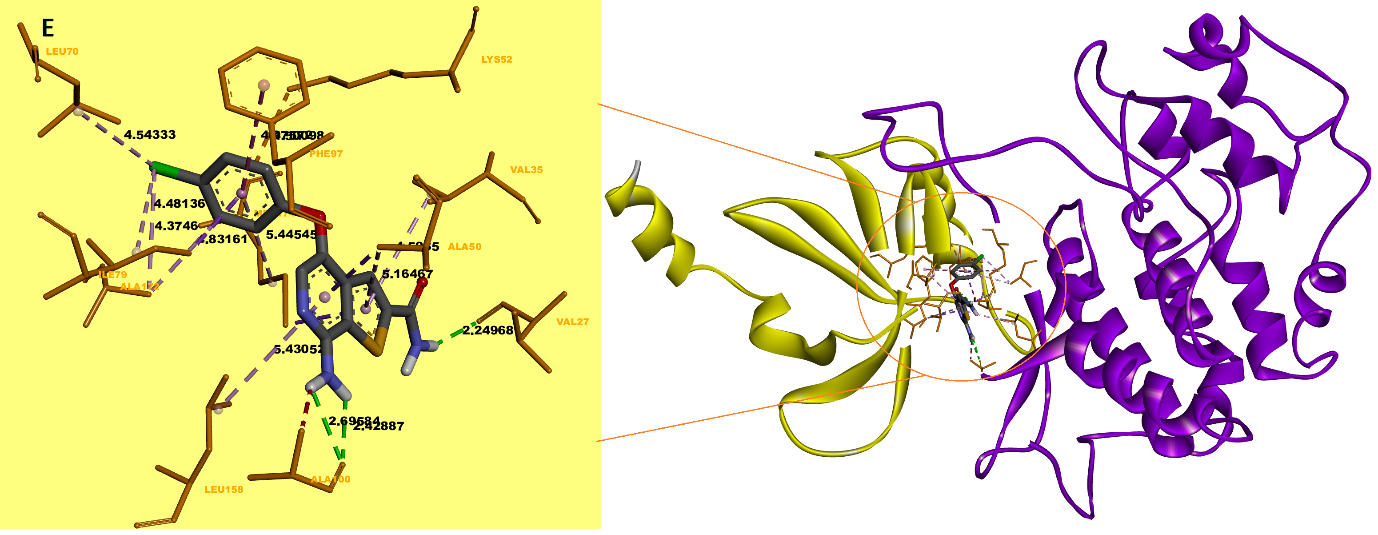


Figure S3 The three-dimensional structures of molecular docking of compound 11 with (A)model-1 (B) D173A (C) D189N (D) T196A (E)T196D. Images were created by Discovery Studio 2021 Client.


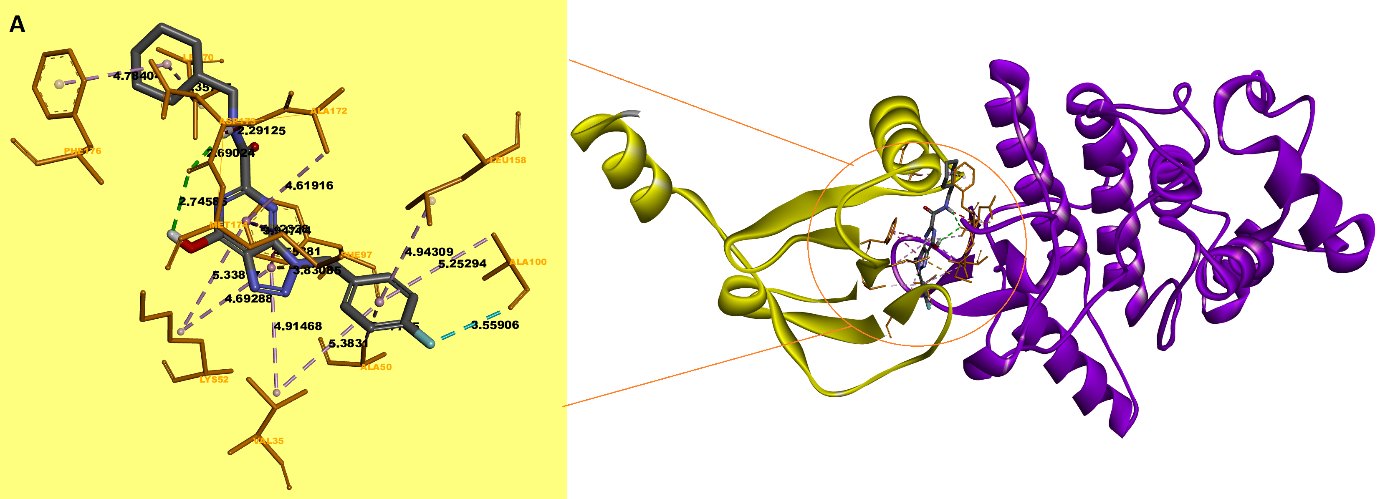


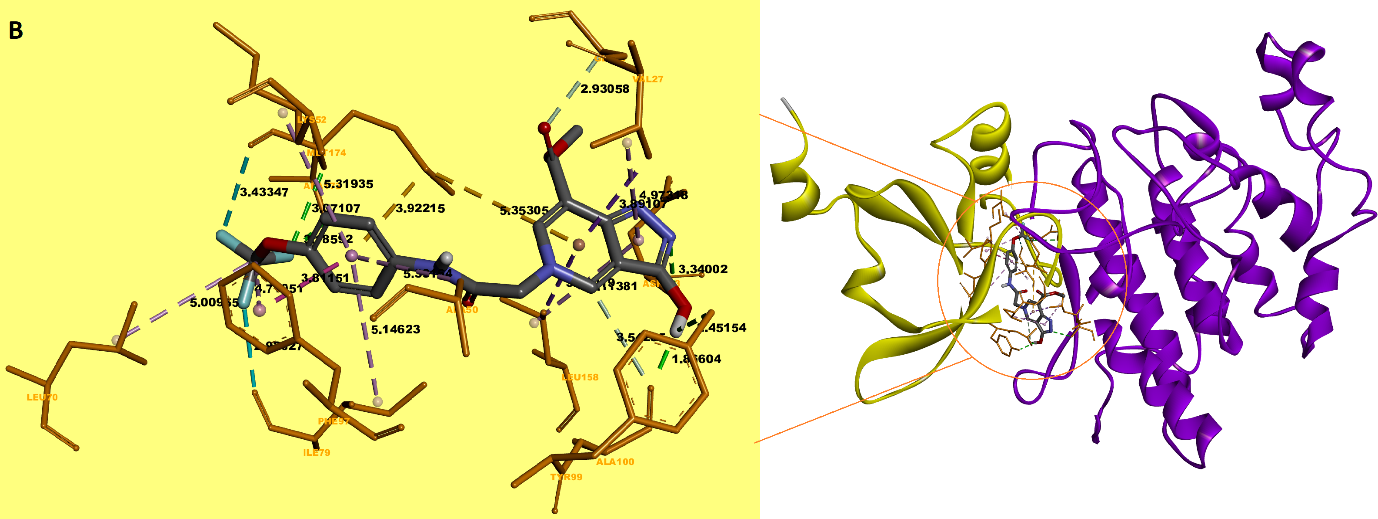


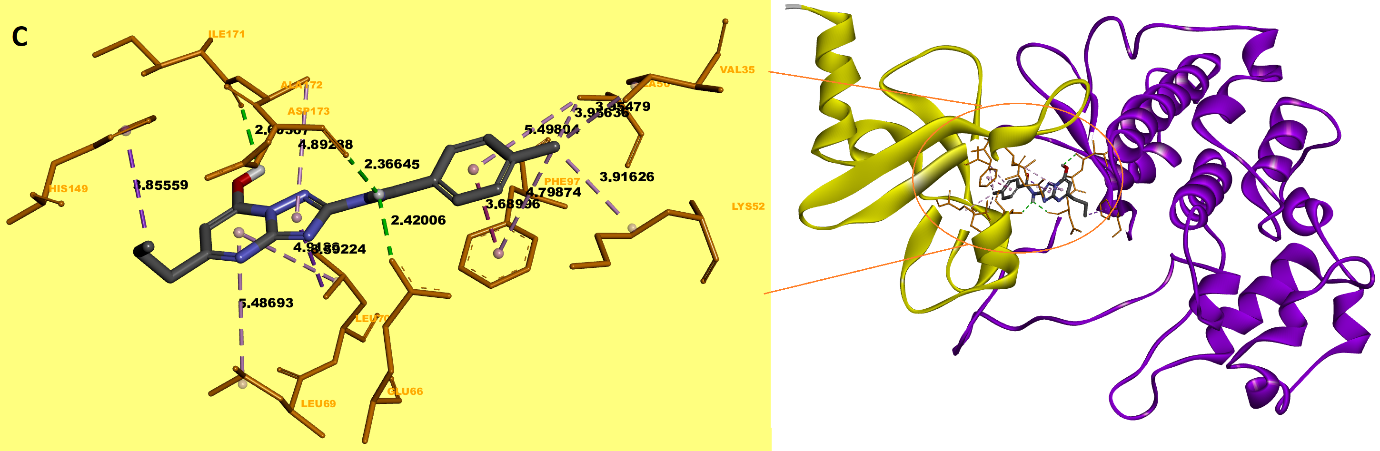


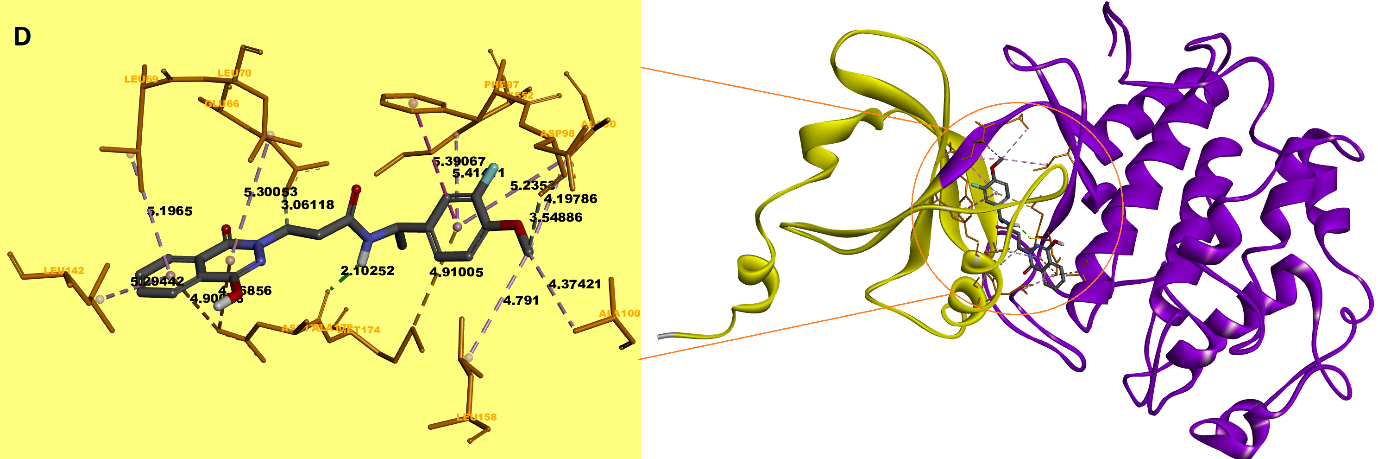


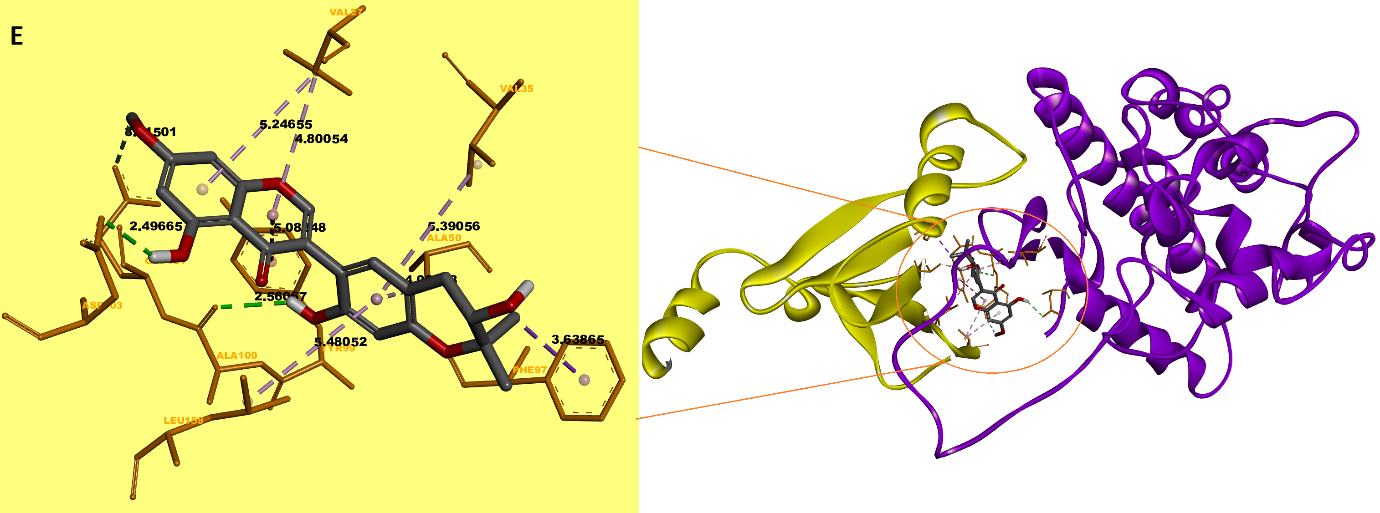


Figure S4 The three-dimensional structures of molecular docking of (A) compound M1 with model-1 (B) compound N7 with D173A (C) compound P7 with D189N (D) compound Q13 with T196A (E) compound R1 with T196D. Images were created by Discovery Studio 2021 Client.
